# Therapeutic Food Development from Maize Grains, Pulses, and Cooking Banana Fruits for the Prevention of Severe Acute Malnutrition

**DOI:** 10.1155/2022/3547266

**Published:** 2022-01-29

**Authors:** Tamiru Yazew

**Affiliations:** Department of Food and Nutritional Sciences, Shambu Campus, Wollega University, Nekemte, Ethiopia

## Abstract

In children under five years of age, severe acute malnutrition is a complex and challenging problem, especially those living in poor communities. Therefore, this study aimed to formulate ready-to-use therapeutic foods from affordable, locally available cereals, pulses, and banana fruits to overcome the problem of severe acute malnutrition. Maize grains, pulses (soybeans), and cooking banana fruits were ingredients used in formulations of ready-to-use therapeutic foods (RUTF). A completely randomized design was done with two replicates. Data were analyzed using analysis of variance. A significant difference existed in the appearance and consistency for RUTF1, RUTF2, and RUTF3 samples (*P* < 0.05). The study findings revealed that the moisture content varied from 6.7 to 13.4%, energy from 513.2 to 642.41 kcal/100 g, protein from 13.9 to 19.34%%, and crude fat from 24.12 to 35.54%. The calcium content ranged from 225 to 302 g/100 mg, iron from 10.34 to 12.26 g/100 mg, and zinc from 10 to 20 g/100 mg. In this study, the phytate content varied from 314.74 to 369.3 *μ*g/g and crude tannin, from 101.36 to 153.25 *μ*g/g. This study concluded that the ingredients used in the RUTF1, RUTF2, and RUTF3 formulations met the standard ready-to-use therapeutic foods. Therefore, it is important to prescribe ready-to-use dietary supplements made from inexpensive, locally available, and culturally acceptable foods to prevent severe acute malnutrition in infants.

## 1. Introduction

Severe acute malnutrition is the most complex stage and affects the growth and brain development of infants and young children [1]. The short- and long-term consequences of severe acute malnutrition are associated with morbidity and mortality [2, 3]. High rates of severe acute malnutrition can also lead to poor child health and nutritional outcomes [4]. Globally, about 16.4 million children under five years of age were affected by severe acute malnutrition [5, 6], and out of whom, 23% accessed ready-to-use therapeutic foods. Approximately 50% of deaths in infants and young children occur due to undernutrition annually [5].

Despite Ethiopia having implemented different nutrition interventions and strategies to reduce the problem of undernutrition, the prevalence of wasting and stunting among children under five was 7% and 37%, respectively [7]. This rate varies from region to region owing to sociocultural and seasonal food insecurity issues [8, 9]. Studies conducted in southern Ethiopia [10] and in the Afar region [11] reported that the prevalence of acute malnutrition among children, which was 14.6% and 16.2%, respectively. This prevalence could also be high because of poverty, food insecurity, floods, and political instability in a nation. However, its prevention has received less attention from the national nutrition policies and programs. Thus, formulations of ready-to-use therapeutic foods from locally available, affordable, energy-dense, macro- and micronutrient-rich foods are the best solution for the prevention of severe acute malnutrition [12, 13]. Food formulations from local and readily available raw materials such as cereals, pulses, and fruits can play an important role in bridging the nutrient and energy gaps during the transition phase. It can also be easily prepared by mothers/caregivers at home.

Maize grain (*Zea mays* L.) is consumed by most rural communities in developing countries [14]. However, it is low in amino acids and micronutrients [15], and this may lead children to micronutrient deficiencies and protein-energy malnutrition. Pulses are highly produced in Ethiopia and are rich in amino acids [16]. Soybean (*Glycine max*) is one of the legume food groups, and it has the highest protein content (around 40%) and 20% of oil than cereals. Cooking banana (*Musa spp*) is also rich in carbohydrates, proteins, vitamins, and minerals [17]. The economic situation in Ethiopia has made it a challenge for many rural households to afford animal source proteins and prevent severe acute malnutrition. In order therefore to prevent severe acute and protein-energy consumption, formulations of ready-to-use therapeutic foods from cheaper plant protein sources such as cereals, pulses and soybeans need to be harnessed for appropriate children feeding. Therefore, this study aimed to formulate affordable, nutrient-dense, and ready-to-use therapeutic foods from maize grain, soybean, and cooking banana fruit for the treatment of severe acute malnutrition in children under five years of age.

## 2. Materials and Methods

### 2.1. Raw Material Collection

Ingredients have been selected for the formulation of ready-to-eat dietary supplements. Ingredients such as yellow maize (*Zea mays* L.), soybeans (*Glycine max*), and banana (*Musa spp)* were collected from Melkassa Agricultural Research Center of Ethiopian Institute of Agricultural Research. Foreign matter, debris, dirt, and other contaminants were removed from the collected samples before further processing begun.

### 2.2. Raw Material Preparation

Malted maize flour samples were prepared according to the previous methods described by [18–20]. Soybean flour samples were also prepared according to the methods described in the previous study [21] and packed in polyethylene bags [22]. The cooking banana fruit flour samples were processed according to a previously described method [23].

### 2.3. Experimental Design

A completely randomized design was used with two replicates.

### 2.4. Development of Ready-To-Use-Therapeutic Foods (RUTF)

Standard guidelines for ready-to-eat dietary supplements were used to formulate ready-to-eat dietary supplements [23]. Three different food samples (RUTF1, RUTF2, and RUTF3) were prepared.

### 2.5. Experimental Procedures

The samples were coded and randomly assigned, and the sample design was randomized for each participant. Ingredient amounts were calculated for RUTF formulations on a dry weight basis. RUTF was added using corn kernels, soybeans, plantain fruits, soybean oil, minerals, and sugar as sweeteners. Three products have been developed based on the specifications of the RUTF World Health Organization (WHO)/United Nations (UN). RUTF1 and RUTF2 had the same ratios of maize grains. RUTF1 sample was formulated from nongerminated ingredients and RUTF2 from germinated ingredients. However, RUTF3 was formulated from the mixtures of germinated and nongerminated raw materials. The ratio of ingredients used for each ready-to-use therapeutic food formulation is given in Table 1.

### 2.6. Proximate and Mineral Analysis

The proximate composition of each flour ingredient and formulated ready-to-use therapeutic foods was determined. The moisture, protein, and crude fat contents were determined using official methods [24]. The Kjeldahl method was used to determine the protein content of the samples [23]. The carbohydrate content was determined using methods reported in a previous study [25]. Minerals such as iron, calcium, and zinc were measured using atomic absorption spectroscopy according to the method described in a previous study [24].

### 2.7. Determination of Antinutritional Factors

The method described in a previous study conducted was used to determine the phytate content [25]. The condensed tannin contents were determined using the previous methods of [26–28].

### 2.8. Sensory Evaluation

Three formulated ready-to-use therapeutic food samples with two replicates were prepared and subjected to sensory evaluation. This evaluation was conducted based on appearance, aroma, taste, and overall acceptability using a five-point hedonic scale, where 1: extremely dislike; 2: moderately dislike; 3: neither like nor dislike; 4: like moderately; and 5: like extremely [29, 30]. The samples were first coded with four digits and randomized for each panelist. Thirty untrained panelists (mothers/caregivers who had infants and young children aged less than two years) participated in the sensory evaluation at the Laboratory of Food and Microbiology, Addis AbabaUniversity.

### 2.9. Statistical Analysis

The data collected from the acceptability test using the questionnaire designed for the samples were analyzed using SPSS version 20.0. Analysis of variance was also done for each response, and the significance test level was set at 5% (*P* < 0.05).

## 3. Results and Discussion

### 3.1. Proximate Composition

This study results revealed that RUTF1 and RUTF2 had higher energies, which were 642.4 and 532.3 kcal/100 g, respectively. The fat content of RUTF2 and RUTF3 had relatively higher than the standard RUTF (see Table 2).

According to the findings of this study, the moisture contents of RUTF1, RUTF2, and RUTF3 were 13.48%, 9.6%, and 6.7%, respectively. The moisture content in this study was higher than that reported in a previous study conducted in Ethiopia, which showed that the moisture content of samples was 8.38%, 5.2%, and 6.8%, respectively [31]. It was also higher than the moisture content of RUTF (2.5%) reported in Nigeria [32]. Similarly, a previous study [33] reported the low moisture content of RUTF. This might be due to the reality that banana fruit may increase the moisture content of the formulated product.

In this study, approximately 19.34%, 16.6%, and 13.9% of protein content accounted for RUTF1, RUTF2, and RUTF3, respectively (see Table 2). This finding was higher than that of the standard RUTF, which was 13.6% [34], and a study conducted in Nigeria [32]. In addition, a study conducted in Kenya reported that the crude protein content of RUTF was 14.47%, 15.43%, and 12.3% for the formulations of P1, P2, and P3, respectively [33]. However, lower protein content was reported in a previous study conducted in Ethiopia [31], in which the protein contents in RTF and PFP were 12.26% and 0.88%, respectively. This was lower than that reported in the current study finding, but PFP was higher. A higher protein content of sorghum (36%) and millet (54%) used for complementary food was reported by [35]. This could be because the nature of ingredients used in product development may vary.

The energy content of the newly formulated products (RUTF1, RUTF2, and RUTF3) was 642.4, 532.3 and 513.2 kcal/100 g, respectively (see Table 2). In this study, the energy content in RUTF1 was higher than those of the standard RUTF, which was 545 kcal/100 g [34], but RUTF2 and RUTF3 were lower energies. This study finding was higher than the previous studies reported in Ethiopia, which showed that the complementary food gross energy varied from 376.30 to 385.56 kcal/100 g [31]. It was also higher than the energy content in formulated RUTF reported in Nigeria [32], which ranged from 520 to 550 kcal/100 g. However, lower energy content (ranged from 359.3 to 380.3 kcal/100 g) than the current study finding was reported by [35]. This could be because the bananas and soybeans added to the current food formulations may provide more energy.

RUTF1, RUTF2, and RUTF3 had 24.12%, 35.54%, and 29.6% crude fat content, respectively. The study finding was lower than those of the standard RUTF, 35.7% [34]. The crude fat content of this study's finding was higher than that reported in the previous study conducted in Ethiopia [31], which ranged from 5.27% to 7.44%. This could be because milk added during formulations of RUTF may increase the level of fat.

The carbohydrate content of the formulated RUTF was 13.4%, 19.0%, and 15.75% for RUTF1, RUTF2, and RUTF3, respectively. This was lower than a finding reported in the previous study [31], in which the carbohydrate content varied from 55% to 70%. Another study conducted in Kenya [33] showed that the carbohydrate content of products: P1, P2, and P3 was 11.64, 39.75, and 23.67%, respectively. In addition, a study conducted in Nepal showed the carbohydrate content of RUTF was higher, 44.73% [36], than the current study's finding.

### 3.2. Mineral Contents (Iron, Calcium, and Zinc)

**Figure 1:**
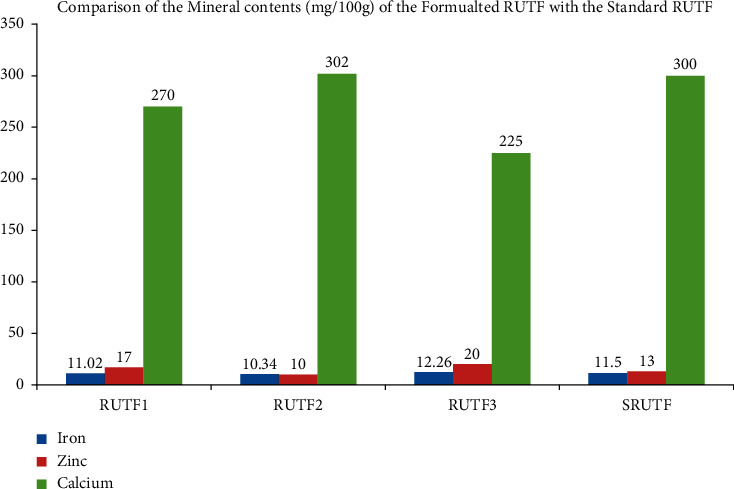
Comparison of the minerals with the standard ready to use therapeutic foods (SRUTF).

The iron content of RUTF1, RUTF2, and RUTF3 was 11.02, 10.34, and 12.26 mg/100 g, respectively. The study findings also reported that the calcium content accounted 270, 302, and 2250 mg/100 g for RUTF1, RUTF2, and RUTF3, respectively (see Figure 1).

The iron content of the RUTF3 (12.26 mg/100 g) was higher than those of the standard ready-to-use therapeutic foods, 11.5 mg/100 g [34] but RUTF1 and RUTF2 were lower. Moreover, another study reported that the iron content of the formulated RUTF was 10–14% [32]. In addition, the study conducted in Nepal also showed that the iron content ofRUTF was 12.27% [36], and higher than the current study's report. According to a study conducted in Ethiopia, the iron content was varied from 0.94 to 38.00 mg/100 g [31].

The zinc content of the formulated RUTF was 17 mg/100 g, 10 mg/100 g, and 20 mg/g, respectively. A similar study in Ethiopia reported that the zinc content of the formulated foods ranged from 4.05–5.58 mg/100 g [31]. A study conducted in Nigeria also showed that the zinc content of the formulated RUTF varied from 11 to 14 mg/100 g [32].

**Figure 2:**
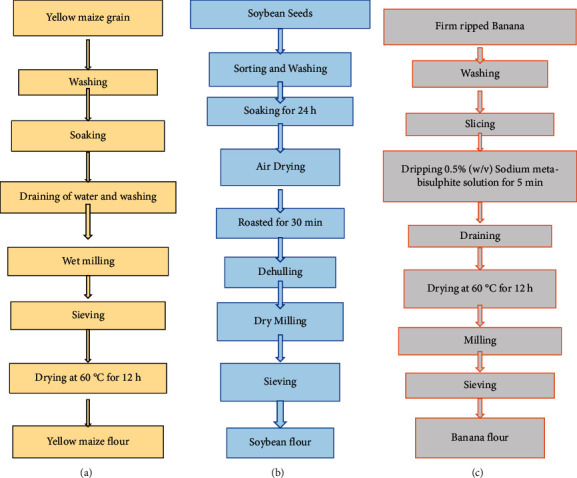
Adopted flow diagrams for the production of maize flour [18–20], soybean flour [18, 21, 22], and banana flour [18, 23].

The calcium content of the formulated RUTF1, RUTF2, and RUTF3 were 270, 302, and 225 g/100 mg, respectively (see Figure 2). The calcium contents in RUTF1 and RUTF3 were lower than in a previous study that reported 300 mg/100 g of calcium [34]. This study finding was lower than that of another study conducted in Nigeria, which reported higher calcium content (300–600 mg/100 g) of RUTF [32]. Another study conducted in Nepal revealed that the calcium content in formulated RUTF was 525 g/100 m, which is higher than the current study's finding [36]. This difference could be due to dry matter loss resulting from respiration during malting.

### 3.3. Antinutritional Factors

The formulated RUTF1 contained 379.30 mg/100 g phytate. The phytate content of formulated products: RUTF1 and RUTF2 had 343.63 and 334.74 mg/100 g, respectively. This study found that the tannin content of RUTF1 and RUTF2 was 173.55 mg/100 g and 102.36 mg/100 g, respectively (see Table 3).

The phytate content of RUTF1, RUTF2, and RUTF3 was 369, 323, and 314 *μ*g/g, respectively. A previous study result also reported that the highest phytate level (295.168 *μ*g/g) was observed in the formulated complementary foods [31]. It was also higher than the phytate content (18.63–175.07 mg/100 g) reported in a previous study [35].

The tannin content was 153.25, 101.36, and 131.41 *μ*g/g in RUTF1, RUTF2, and RUTF3, respectively (see Table 3). A similar study reported that the highest tannin content in the porridge prepared from FM2 [31]. The current study finding was higher than that of another study conducted in Ethiopia, which reported the tannin content ranged from 0.84 to 42.89 mg/100 g [35].

### 3.4. Sensory Evaluation

According to the findings of this study, the overall averages based on attributes such as appearance, consistency, sweetness, and flavor of RUTF1, RUTF2, and RUTF3 were 26, 27, and 26, respectively (see Table 4).

Regarding the sensory evaluation, this study found that there was a statistical significance among the three products in terms of appearance and consistency (see Table 4). The different sets of attributes that lead to the most preferred highest and least liked products may be due to different cultural backgrounds, experiences, attitudes, and habits of respondents [37]. Other study results showed that the sensory acceptability of all formulated complementary foods scored better value in terms of appearance and overall acceptability [31].

## 4. Conclusion

This study findings concluded that there was a statistical significance in appearance and consistency among the three formulated ready-to-use therapeutic foods. In the updated current study, the protein content of all three formulations (RUTF1, RUTF2, and RUTF3), the energy of RUTF1, the fat of RUTF2, the iron of RUTF3, the zinc of RUTF3, and the calcium of RUTF2 are higher than the standard finished product. In addition, current studies have found that ready-to-eat medicated foods with phytate and tannin content are lower than non-germinated samples. Therefore, it is important to use a variety of food processing methods to increase the nutrient content of ready-to-eat dietary supplements to overcome the problem of severe acute malnutrition among infants and young children.

## Figures and Tables

**Table 1:** Ratio and ingredients used in the formulations of ready-to-use therapeutic foods.

Formulation	Ingredients						
	Maize (g)	Soybean (g)	Banana (g)	Soy oil (g)	Sugar (g)	Mineral (mg)	Total
RUTF1	28	20	15	15	20	2	100
RUTF2	28	25	0	20	25	2	100
RUTF3	0	60	0	10	28	2	100

RUTF1, ready-to-use therapeutic food1; RUTF2, ready-to-use therapeutic food2; RUTF3, ready-to-use therapeutic food3.

**Table 2:** Proximate composition of ready-to-use therapeutic foods.

Formulation	Major nutrients and moisture				
	Protein (%)	Energy (kcal/100 g)	Carbohydrate (%)	Fat (%)	Moisture (%)
RUTF1	19.34	642.4	13.4	24.12	13.4
RUTF2	16.6	532.3	19.0	35.54	9.6
RUTF3	13.9	513.2	15.75	29.6	6.7
SRUTF	13.6	545	-	35.7	-

RUTF1, ready-to-use therapeutic food1; RUTF2, ready-to-use therapeutic food2; RUTF3, ready-to-use therapeutic 3; SRUTF, standard ready -to–use- therapeutic food.

**Table 3:** Anti-nutritional factors of ready-to-use therapeutic foods.

Formulation	Types of antinutritional factors	
	Phytate (*μ*g/g)	Tannin (*μ*g/g)
RUTF1 (NG)	369.30 ± 0.39a	153.25 ± 0.35a
RUTF2 (G)	323.63 ± 0.29c	101.36 ± 0.46c
RUTF3 (MNGG)	314.74 ± 0.42 b	131.41 ± 0.11b

RUTF1, ready-to-use therapeutic food1; RUTF2, ready-to-use therapeutic food2; RUTF3, ready-to-use therapeutic food3; SRUTF, standard ready-to-use therapeutic food; NG, nongerminated; G, germinated; MNGG, mixtures of nongerminated and germinated. Similar letters indicate that there are no significant differences in the three products, and different letters indicate that there is a significant difference in the products.

**Table 4:** Average and mean acceptability scores of the formulated ready-to-use therapeutic foods.

Formulation	Mean acceptability scores				
	Appearance	Consistency	Taste	Flavour	Overall
RUTF1 (mean)	7 (7.21)	6 (6.34)	6 (6.13)	7 (6.45)	26 (26.13)
RUTF2 (mean)	7 (7.42)	6 (6.23)	7 (7.52)	7 (6.44)	27 (27.61)
RUTF3 (mean)	7 (7.64)	6 (7.25)	6 (7.18)	7 (7.23)	26 (29.30)

RUTF1, ready-to-use therapeutic food1; RUTF2, ready-to-use therapeutic food2; RUTF3, ready-to-use therapeutic food3.

## Data Availability

Datasets that support this are available from the corresponding author upon reasonable request.
